# Comprehensive application of artificial intelligence in preserved ratio impaired spirometry: A systematic literature review

**DOI:** 10.1371/journal.pone.0353021

**Published:** 2026-09-16

**Authors:** Qian Wu, Hui Guo, Ruihan Li, Jinhuan Han, Zhen Zhang, Ayajiang Jingesi

**Affiliations:** Department of Medical Imaging Center, The Fourth Clinical Medical College of Xinjiang Medical University, Urumqi, Xinjiang, China; UFSJ: Universidade Federal de Sao Joao del-Rei, BRAZIL

## Abstract

**Background:**

Artificial intelligence (AI) has expanded into respiratory disease diagnosis, subtyping, and prognosis, enabling early detection and precision care. However, AI applications in Preserved Ratio Impaired Spirometry (PRISm) remain nascent. This study analyzes this gap to guide future research.

**Methods:**

A systematic review was conducted to analyze the application of AI in PRISm, searching across PubMed, Cochrane Library, Web of Science, Ovid Medline, Scopus and Embase.

**Results:**

A total of eleven studies were included, all of which focused on diagnostic and classification tasks. Among these, three utilized radiomics models, four employed machine learning algorithms, two integrated machine learning with radiomics, and two applied deep learning approaches. Nine studies were published within the past two years, with results demonstrating the high performance and developmental potential of AI technologies in this domain. AI research on PRISm spans multiple disciplines, including exhaled metabolomics, environmental exposure assessment, radiomics, and deep learning.

**Conclusion:**

Existing studies have preliminarily validated the technical feasibility of artificial intelligence for the early identification of PRISm from multiple perspectives, including imaging, metabolism, and environmental exposure. Modeling strategies that integrate multi-source data have demonstrated superior discriminatory performance compared to single-modality approaches. In the future, with the integration and sharing of multicenter data under privacy-compliant conditions, coupled with the continuous evolution of algorithm architectures toward enhanced generalizability, AI applications for PRISm are expected to transition from static identification to dynamic early warning, thereby providing more robust technical support for precise risk stratification of this condition.

## 1. Introduction

Preserved Ratio Impaired Spirometry (PRISm) is increasingly recognized in epidemiological and clinical contexts as a potential prodromal state for chronic obstructive pulmonary disease (COPD) and interstitial lung pathologies. This spirometric pattern independently predicts COPD progression and correlates with all-cause mortality, respiratory-specific mortality, cardiovascular morbidity, diabetes mellitus, and chronic kidney disease [1,2]. While direct neurological associations remain unconfirmed, preliminary evidence indicates possible dementia risk elevation in PRISm populations [3]. Neuroimaging studies reveal structural alterations in PRISm patients, including reduced cortical surface area in the paracentral lobule and precuneus, alongside decreased gray matter thickness in the inferior parietal lobe and temporal pole [4]. These regions mediate essential cognitive functions including visual processing, memory consolidation, linguistic capacity, and emotional regulation, suggesting early neurological involvement in PRISm pathogenesis. PRISm cohorts demonstrate significantly impaired cognitive performance and elevated lacunar infarction prevalence compared to healthy controls, mirroring deficits observed in Chronic Obstructive Lung Disease(GOLD) 2–4 COPD patients [5]. Notably, lung cancer patients with concurrent PRISm exhibit reduced overall survival compared to both COPD patients and lung cancer patients with normal pulmonary function [6,7]. In real-world clinical practice, approximately 25% of patients receiving COPD management fail to meet GOLD spirometric criteria, with 13% classified as pre-COPD and 14% as PRISm [8, 9].

Diagnostic evaluation of PRISm primarily involves spirometric assessment, complemented by chest computed tomography, pulse oximetry, and biomarker analysis. However, conventional spirometry interpretation remains subject to operator variability, constraining its capacity to delineate disease heterogeneity. Artificial intelligence (AI) demonstrates potential to overcome these limitations through multimodal data synthesis and advanced pattern recognition algorithms.

Existing scholarly reviews on PRISm have predominantly emphasized clinical characterization [10,11]. A 2025 synthesis examined PRISm through clinical, imaging, and AI perspectives but offered limited technical depth regarding AI implementation [12]. In the field of pulmonary diseases, particularly COPD, AI technology has been extensively integrated into various clinical tasks, including diagnosis, predictive modeling, disease staging, and outcome assessment [13]. The confluence of AI and PRISm research remains in an embryonic stage of development. This systematic review comprehensively synthesizes current evidence to delineate research advancements, methodological constraints, and strategic priorities for advancing this evolving field.

## 2. The epidemiology and pathophysiological mechanisms of PRISm

Two decades ago, researchers identified patients presenting with respiratory symptoms (e.g., dyspnea) and radiological abnormalities despite normal spirometry byconventional COPD criteria. The COPDGene study formally established the term PRISm a decade later, laying the foundation for subsequent research. PRISm was defined by the COPDGene study in 2013 and formally recognized in the 2023 GOLD guidelines. It is characterized by post-bronchodilator FEV1/FVC ≥ 0.7 and either FEV1 or FVC < 80% predicted, suggesting its potential role as a pre-COPD state [14]. Subtype analyses indicate that non-restrictive PRISm, which meets neither traditional obstructive nor restrictive criteria, is independently linked to COPD progression [15]. The global prevalence of PRISm ranges from 4.49% to 24.4%, with notably higher rates observed in low- and middle-income countries [16–19]. Both preclinical COPD (defined by normal spirometry with respiratory symptoms or structural-functional abnormalities) and PRISm independently predict COPD progression [20,21]. PRISm demonstrates dynamic disease trajectories [22], with progression risks influenced by advanced age, male gender, abnormal BMI, heavy smoking history, reduced DLCO, small airway dysfunction, and persistent PRISm status [23,24].

The pathological mechanisms underlying PRISm involve systemic inflammation (elevated IL-6, IL-8, and CRP levels), lung structural abnormalities, and metabolic dysregulation such as oxidative stress [25,26]. Although no targeted therapies currently exist, early identification of high-risk subgroups (e.g., non-restrictive PRISm) and interventions such as lung function monitoring and risk factor control are critical for delaying COPD progression. Significant gaps remain in understanding the mechanisms and developing therapeutic strategies, necessitating further research.

## 3. Diagnostic examinations for PRISm

### 3.1. Pulmonary function test

#### 3.1.1. Forced spirometry.

The patient is instructed to take a maximal deep inhalation followed by a forced rapid exhalation to measure forced expiratory volume in the first second (FEV₁) and forced vital capacity (FVC). One diagnostic criterion for PRISm is a post-bronchodilator FEV₁/FVC ratio ≥0.7, with either FEV₁% predicted or FVC% predicted <80%.

#### 3.1.2. Body plethysmography.

This test evaluates pulmonary volume status and is critical for differentiating restrictive and obstructive ventilatory defects. PRISm patients typically exhibit normal total lung capacity (TLC). However, they may present with a restrictive ventilatory defect pattern (normal TLC but reduced FVC and FEV₁), where both FVC% predicted and FEV₁% predicted are decreased while maintaining a preserved FEV₁/FVC ratio.

### 3.2. Imaging examination

#### 3.2.1. High-resolution computed tomography (HRCT).

HRCT delivers superior anatomical resolution for characterizing fine structural details, critically valuable in identifying emphysema, interstitial lung disease, pleural thickening, and effusions. HRCT enhances diagnostic precision by detecting subradiographic early pathological changes, significantly contributing to PRISm differentiation and comprehensive evaluation [27,28].

### 3.3. Biomarker

#### 3.3.1. Exhaled volatile organic compounds (VOCs) detection.

Exhaled VOCs may serve as effective biomarkers for diagnosing various respiratory diseases and can also reliably reflect disease severity and phenotypes. Key advantages include non-invasive sampling, repeatable measurements, and applicability for large-scale screening.

#### 3.3.2. Blood tests.

Detect inflammatory markers in the blood, such as C-reactive protein (CRP) and interleukin-6 (IL-6), to evaluate systemic inflammatory status. Some PRISm patients may exhibit chronic inflammatory responses, and elevated levels of these biomarkers may correlate with disease progression and prognosis [29,30].

### 3.4. Other examinations

#### 3.4.1. Impulse oscillometry (IOS).

Based on the Forced Oscillation Technique (FOT), this method applies exogenous oscillatory pressure waves to the respiratory system while simultaneously measuring airflow and pressure changes to calculate airway resistance (R, reflecting resistive airway properties) and reactance (X, associated with lung elastic recoil). It requires no active patient cooperation (e.g., forced breathing maneuvers), making it particularly suitable for individuals with respiratory limitations. Studies indicate that a percentage predicted R5–R20 ≥ 120% serves as an effective diagnostic threshold for PRISm, demonstrating high sensitivity and specificity [31,32]. Furthermore, combined analysis of resonant frequency (Fres) and reactance area index (AX) provides a comprehensive assessment of airway and lung tissue mechanical abnormalities, offering supplementary diagnostic insights for PRISm.

#### 3.4.2. Electrical impedance tomography (EIT).

As a non-invasive and radiation-free pulmonary imaging technique, EIT evaluates ventilation heterogeneity by detecting variations in electrical impedance distribution. Studies demonstrate that COPD patients exhibit the highest proportion of lung regions with an FEV1/FVCEIT ratio <0.7, while PRISm patients show a significantly higher percentage of abnormal regions compared to healthy controls, with marked statistical differences [33]. This highlights EIT's potential for early detection of PRISm and other pulmonary disorders.

#### 3.4.3. Bronchoprovocation and bronchodilation tests.

Bronchial provocation tests assess airway hyperresponsiveness to stimuli, aiding in the diagnosis of latent airway dysfunction in PRISm. Conversely, bronchodilator reversibility testing evaluates airway reversibility, with some PRISm patients demonstrating post-bronchodilator lung function improvements—a critical feature for guiding personalized therapeutic strategies [34,35].

## 4. The application of AI in preserved ratio impaired spirometry

### 4.1. Overview of Artificial Intelligence

As an emerging interdisciplinary technology, AI in the medical field integrates complex omics data with additional layers (including imaging and electronic health data) and performs quantitative analysis on large datasets. The concept of AI was formally introduced at the first AI conference in the United States in 1956 and has gradually gained widespread recognition. The three core technical directions of AI are radiomics, machine learning, and deep learning.

Radiomics is the discipline of extracting large-scale quantitative features from medical images and identifying potential disease patterns by analyzing these features. It typically involves multiple steps: image acquisition, preprocessing, segmentation of regions of interest, feature extraction and selection, and finally combining these features with other clinical data to build models for diagnosis, prognosis prediction, or treatment evaluation [36,37].

Machine Learning is a technology that enables computer systems to automatically learn and improve from data through algorithms. In medical imaging, machine learning generally requires feature extraction to convert images into quantitative features, which are then used for classification or regression tasks. For example, texture or shape features extracted from images can train models to distinguish between normal and pathological tissues.

Deep Learning, a subset of machine learning, utilizes artificial neural networks (especially deep neural networks) to autonomously learn complex feature representations from data. It is widely applied in medical imaging, with convolutional neural networks (CNN) being the most common architecture. Its strength lies in automatic feature extraction without manual design, robust modeling capabilities for large-scale datasets, and the ability to uncover intricate patterns, achieving superior performance in tasks like image classification, object detection, and segmentation.

The deep integration of AI in medical imaging diagnostics enables automatic lesion detection through analysis of X-ray, CT, and MRI scans [38,39]; constructs disease prediction models by synthesizing multi-source data to guide personalized treatment [40,41]; assists in radiotherapy target delineation and surgical planning to optimize therapeutic outcomes [42]; and accelerates medical research and drug development, thereby driving the advancement of precision medicine [43–45].

### 4.2. Purpose and scope of the review

This review examines the current applications of AI in PRISm research. By integrating multinational cohort data and clinical case studies, it analyzes AI's potential in data integration, phenotypic subtyping, risk prediction, and treatment optimization. The review also addresses current technical limitations and proposes future research directions to guide advancements in this field.

### 4.3. Methods

This is a systematic review registered with the PROSPERO International Prospective Register of Systematic Reviews (CRD420251052933). This study was conducted in accordance with the PRISMA guidelines; relevant information and search strategies are detailed in the supporting information(the prisma_2020_checklist and the Search). We searched PubMed, Web of Science, Embase, Cochrane Library, Ovid Medline and Scopus for studies published up to March 15, 2026, using the keywords “Deep Learning,” “Machine Learning,” “Radiomics,” and “PRISm” with no restrictions on study type, language, or country. These databases currently represent the largest and most widely utilized medical databases available, capable of fulfilling diverse research requirements. Review articles and conference abstracts were excluded. Study selection and data extraction were independently performed by two radiologists (Wu and Li), with a third researcher (Han) resolving discrepancies.

#### 4.3.1. Included.

Studies will be included in the research if they meet the following inclusion criteria: the participants meet the criteria of a normal FEV1/FVC ratio (≥0.7) after inhaling a bronchodilator, but with FEV1 (or FVC) below 80% of the predicted value; studies will report application of AI. The AI models categorized in this study comprise three distinct types: radiomics, machine learning, and deep learning.

#### 4.3.2. Excluded.

Studies will be excluded if they meet the following exclusion criteria: the participants don't meet the criteria of a normal FEV1/FVC ratio (≥0.7) after inhaling a bronchodilator, but with FEV1 (or FVC) below 80% of the predicted value; studies which have not applied any type of AI; meeting summaries or overviews.

### 4.4. Results

#### 4.4.1. Study selection.

**Fig 1:**
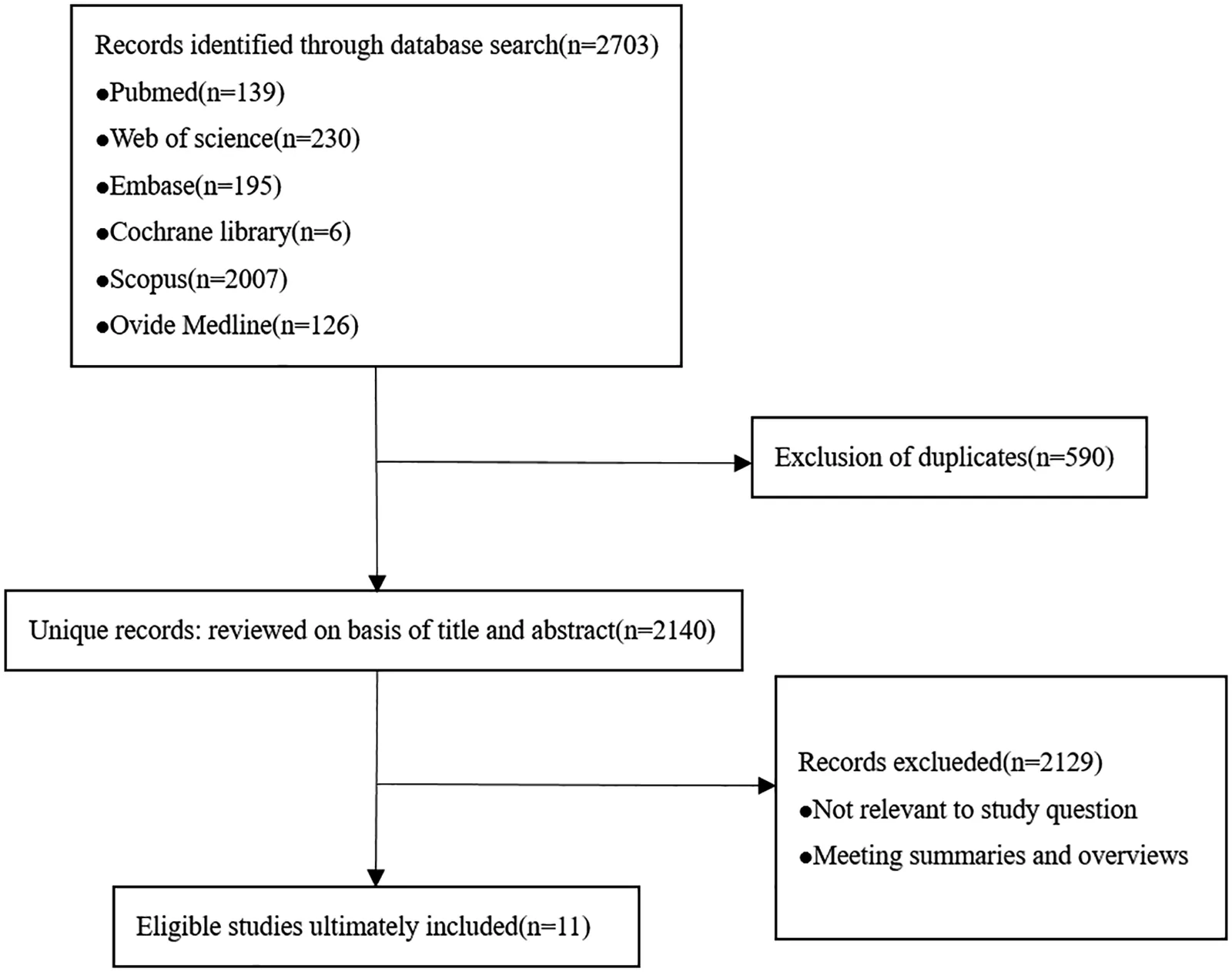
Study selection process.

A total of 2703 papers were obtained from the database search, and a total of 590 papers were excluded as duplicates using EndNote 20. After reviewing titles, abstracts, and full texts to exclude irrelevant studies, and reviewing the eligible studies in the references of the included studies and relevant reviews, 11 papers were finally retained. The specific screening process for this study was shown in Fig 1.

#### 4.4.2. Studies’ quality assessment.

**Fig 2:**
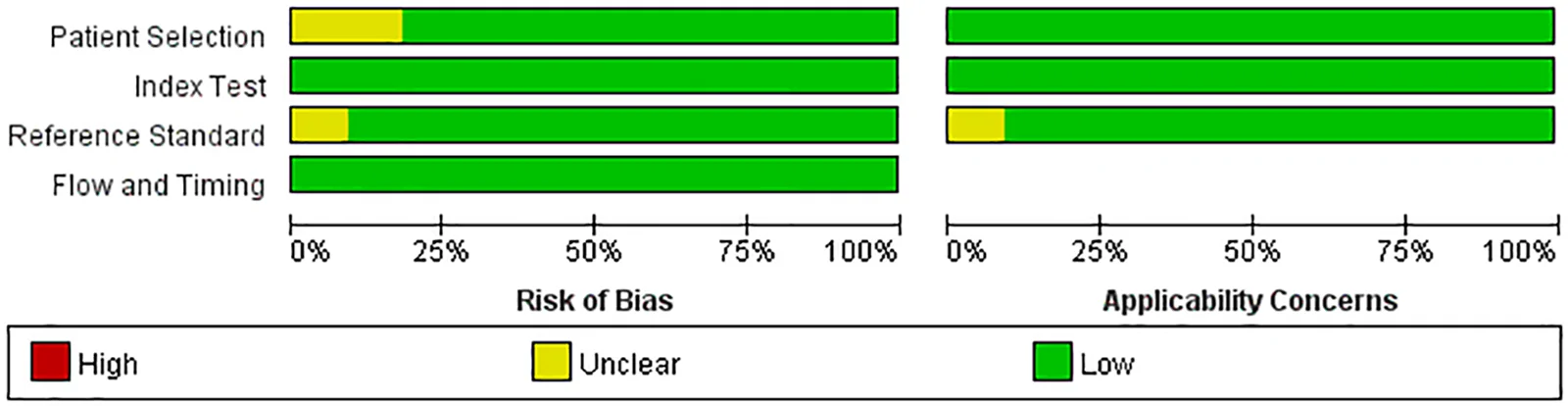
Quality assessment.

Since all eleven included studies were diagnostic in design, the Quality Assessment of Diagnostic Accuracy Studies 2 (QUADAS-2) tool was applied to evaluate the methodological quality using RevMan 5.4 software. The results of this quality appraisal were presented in Fig 2. The tool comprises four domains: patient selection, index test, reference standard, and flow and timing; each domain is rated as having a high, low, or unclear risk of bias. In the patient selection domain, two studies were deemed unassessable for risk of bias because the datasets failed to describe whether the cohorts were randomly or consecutively sampled and falied to specify the time frame. In the reference standard domain, one study was deemed unassessable for both risk of bias and applicability because the inclusion criteria for patients in the dataset were not described.

#### 4.4.3. Study characteristics.

Given the limited number of included studies, this study conducted a systematic review rather than a meta-analysis. The characteristics of the included studies are summarized in Table 1. Among the eleven studies, ten originated from China and one from Canada. Two studies were initiated in 2024, while the remaining seven were conducted in 2025 and 2026. Three studies employed radiomics approaches, four utilized machine learning algorithms, two applied deep learning techniques, and two integrated machine learning with radiomics. Four studies used datasets from public repositories, whereas the remaining seven relied on hospital-based datasets. All eleven studies were diagnostic in nature.

**Table 1:** Application of AI models in PRISm.

Author	Country	Year	Sample Size	Model Type	Study Type	Results
**Zhou et al [47] (1)**	China	2024	1066	Radiomics	Diagnostic	radiomics and clinical model(AUC: 0.82, 0.77, 0.84; ACC: 0.75, 0.70, 0.73; SE: 0.68, 0.73,0.90; SP: 0.83, 0.68, 0.56)
**Zhou et al [48] (2)**	China	2024	1481	Radiomics	Diagnostic	radiomics and clinical model(AUC: 0.79, 0.77, 0.70; SE: 0.75, 0.64,0.64; SP: 0.69, 0.79, 0.68)
**Lukhumaidze et al [49]**	Canada	2025	596(CanCOLD)	Machine learning+ Radiomics	Diagnostic	stable PRISm and stable control(AUC: 0.84, Recall: 0.92, F1 score: 0.54) stable PRISm and stable COPD(AUC: 0.92, Recall: 0.87, F1 score: 0.60)
**Tian et al] [52]**	China	2025	367	Machine learning	Diagnostic	Prism and healthy control(AUC: 0.78, SP: 0.69, SE: 0.87) Prism and asthma(AUC: 0.74, SP: 0.82, SE: 0.66)
**Deng et al [53] (1)**	China	2025	2616(Nhanes)	Machine learning	Diagnostic	LightGBM(AUC: 0.84, ACC: 0.72, Recall: 0.91, SP: 0.68, PPV: 0.36, NPV: 0.98)
**Deng et al [54] (2)**	China	2025	8836(Nhanes)	Machine learning	Diagnostic	LightGBM(AUC: 0.81 ACC: 0.74 SE: 0.77 SP: 0.73)
**Deng et al [55] (3)**	China	2025	1363(Nhanes)	Machine learning	Diagnostic	CatBoost(AUC:0.83 ACC: 0.74 SE: 0.85 SP: 0.71 PPV: 0.44 NPV: 0.94 F1 score: 0.58)
**Fu et al [56]**	China	2025	270	Machine learning+ Radiomics	Diagnostic	Clinical-Dual-phase-radiomic model(AUC:0.81 ACC: 0.63 SE:0.58 SP: 0.93 PPV: 0.98 NPV: 0.25)
**Ma et al [57]**	China	2025	1018	Radiomics	Diagnostic	Lung fusion model(AUC: 0.83 ACC: 0.77 SE: 0.76 SP: 0.78 PPV: 0.64 NPV: 0.86)
**Wu et al [58] (1)**	China	2026	1624	Deep learning	Diagnostic	DenseNet(AUC: 0.85 ACC: 0.79 Precision: 0.58 Recall: 0.50 F1score:0.54)
**Wu et al [59] (2)**	China	2026	1918	Deep learning	Diagnostic	PulmoClass-3DAtt(AUC: 0.86 ACC: 0.87 SE: 0.42 SP: 0.97 Precision: 0.72 F1 score: 0.53)

Note: (1), (2), and (3) denote distinct studies by the same first author.

### 4.5. Disease diagnosis and differential diagnosis

Imaging investigations into PRISm remain in their formative stages. AI demonstrates diagnostic efficacy through identification of nuanced structural signatures on high-resolution computed tomography (HRCT), particularly bronchial wall thickening patterns, enabling differentiation between PRISm and COPD [46].

Zhou et al. [47] established a CT-derived radiomics classification model to distinguish PRISm (n = 548) from COPD (n = 518) across multicenter cohorts. Leveraging 1,218 quantitative imaging features extracted from volumetric CT data with LASSO regularization, the model attained AUC values of 0.82 (training cohort), 0.77 (internal validation), and 0.80 (external validation). Multimodal integration with clinical parameters enhanced external validation performance (AUC = 0.84), with methodological reproducibility confirmed in subsequent investigations [48].

Lukhumaidze et al. [49] implemented three machine learning architectures (logistic regression, random forest, support vector machine) incorporating 34 quantitative CT metrics (including %TLCCT and %GG+Reticulationtexture) and 102 radiomic descriptors (e.g., GLDZMZDV) to discriminate stable PRISm (n = 22), healthy controls (n = 274), and stable COPD (n = 300). Due to sample size limitations, pairwise comparative analysis revealed RF models combining clinical, imaging, and radiomic features achieved peak discriminative performance: AUC 0.84 (PRISm vs controls) and 0.92 (PRISm vs COPD), significantly surpassing clinical-only models (p < 0.05). SHAP interpretability analysis identified characteristic PRISm airway remodeling patterns: increased wall thickness (Pi10 = 3.2 ± 0.4 mm), reduced lumen area (4.1 ± 1.2 mm²), and diminished airway count (18.3 ± 3.1) – concordant with COPDGene consortium findings.

Previous studies have demonstrated that exhaled volatile organic compounds (VOCs) may serve as effective biomarkers for diagnosing various respiratory diseases, including chronic obstructive pulmonary disease (COPD), lung cancer, asthma, and COVID-19 [50]. Exhaled VOCs not only facilitate the differentiation of distinct disease entities but also reliably reflect disease severity and phenotypic variations [51]. Endogenous VOCs in exhaled breath, generated through diverse metabolic processes and transported to alveoli via blood-gas exchange, may reflect distinct pathophysiological conditions in patients. Current analytical techniques for exhaled VOC detection include gas chromatography-mass spectrometry (GC-MS), proton transfer reaction mass spectrometry (PTR-MS), and electronic nose (eNose) systems. However, these methods are constrained by technical complexity, high costs, operational inconvenience, and prolonged analytical durations. Tian et al. [52] employed non-invasive breathomics profiling to identify VOC signatures for distinguishing chronic respiratory diseases and subsequently developed machine learning models for early detection of PRISm, COPD, and asthma. The study enrolled 367 patients from a university-affiliated hospital, including 72 PRISm cases, utilizing a portable micro gas chromatography (micro-GC) device for real-time automated exhaled VOC analysis. Five machine learning algorithms—logistic regression, support vector machine (SVM), random forest, extreme gradient boosting (XGBoost), and K-nearest neighbors (KNN)—were implemented for disease classification. Key findings revealed that the SVM classifier achieved optimal performance in distinguishing PRISm from healthy controls, with an area under the curve (AUC) of 0.78 ± 0.01, specificity of 69%, and sensitivity of 87%. For differentiating asthma from PRISm, the logistic regression model attained an AUC of 0.74 ± 0.02, specificity of 82%, and sensitivity of 66%. Furthermore, nine VOCs were identified as discriminative markers between PRISm and healthy populations. Five of these VOCs demonstrated substantial concordance with COPD-associated compounds, albeit with divergent concentration trends. Notably, p-xylene levels were significantly reduced in COPD patients but elevated in PRISm cases compared to healthy controls, suggesting potential pathophysiological relevance. Reduced acetone levels in PRISm patients may partially explain diminished expiratory flow rates. This pioneering study represents the first application of portable micro-GC in investigating exhaled VOC profiles across COPD, PRISm, asthma, and other chronic respiratory conditions. The findings underscore the developmental potential of this field, with the integration of AI technology enhancing diagnostic efficiency and scalability.

Deng et al.[53] utilized data from the 2007–2012 National Health and Nutrition Examination Survey (NHANES) in the United States, comprising 2,616 participants, and systematically evaluated the associations between eight blood-based volatile organic compounds (VOCs) and PRISm using five analytical approaches: multivariate logistic regression, weighted quantile sum regression, quantile g-computation, Bayesian kernel machine regression, and machine learning algorithms. The results demonstrated that, for machine learning prediction, the study employed SMOTE oversampling to address class imbalance and constructed ten models, including Light Gradient Boosting Machine (LightGBM), Categorical Boosting (CatBoost), and Extreme Gradient Boosting (XGBoost). Among these, LightGBM achieved the highest area under the receiver operating characteristic curve (AUC-ROC) of 0.84, and SHAP analysis revealed that race, body mass index (BMI), and 1,4-dichlorobenzene were the three most influential features. In a subsequent study, Deng et al.[54] conducted a mixed-methods investigation into the association between dietary fatty acid intake and PRISm. For predictive modeling, the study applied six algorithms: LightGBM, Decision Tree, Multi-Layer Perceptron, Naive Bayes, k-Nearest Neighbors, and Support Vector Machine. LightGBM achieved optimal performance with an accuracy of 0.74. SHAP analysis indicated that race, BMI, and butyric acid were the most influential predictors. Additionally, Deng et al.[55] examined the association between endocrine-disrupting chemicals and PRISm in another study. For the machine learning component, the Least Absolute Shrinkage and Selection Operator (LASSO) was employed for feature selection, and seven models—including CatBoost, XGBoost, and Random Forest—were constructed. CatBoost exhibited the best performance, with an AUC-ROC of 0.83. SHAP analysis further confirmed that race and monoisobutyl phthalate were the variables contributing most significantly to demographic and environmental exposure factors, respectively.

Fu et al.[56] conducted a prospective study utilizing dual-phase CT radiomics and machine learning for PRISm identification. The study comprised 270 participants. Radiomic features were extracted from inspiratory, expiratory, and dual-phase breath-hold chest CT images. Clinical models, radiomic models, and fusion models (integrating radiomic features with clinical variables) were constructed. The researchers systematically compared ten machine learning algorithms, including logistic regression, Naive Bayes, support vector machine, random forest, extreme gradient boosting (XGBoost), light gradient boosting machine (LightGBM), and multi-layer perceptron. The results demonstrated that expiratory-phase and dual-phase fusion models based on logistic regression achieved area under the receiver operating characteristic curve (AUC-ROC) values of 0.82 and 0.81, respectively, in the external validation set; incorporation of inspiratory-phase data did not significantly enhance model performance. Based on these findings, the researchers recommend prioritizing single-phase expiratory CT combined with clinical features and logistic regression for efficient PRISm identification in clinical practice, thereby facilitating early diagnosis and intervention while minimizing radiation exposure.

Ma et al.[57] developed a three-class classification model integrating clinical variables with chest CT radiomics to differentiate normal lung function, PRISm, and COPD. The study comprised 797 participants. Five predictive models—clinical, airway, lung, airway fusion, and lung fusion—were constructed using multinomial logistic regression. The results demonstrated that the lung fusion model, which integrated age, sex, body mass index, and whole-lung radiomic features, exhibited optimal performance. In the external validation set, it achieved area under the receiver operating characteristic curve (AUC-ROC) values of 0.94, 0.83, and 0.90 for the normal, PRISm, and COPD groups, respectively, with an overall accuracy of 83.59%. This study demonstrates that whole-lung radiomic features outperform airway tree features alone in capturing early structural alterations in PRISm, thereby providing a robust tool for precise radiological classification of chronic airway diseases.

Wu et al.[58] developed a multimodal multi-task learning framework for simultaneous automated detection and classification of chronic obstructive pulmonary disease (COPD). This retrospective study enrolled 2,320 participants, with chest CT images fused with clinical variables serving as model inputs. A 3D convolutional neural network was employed as the backbone of the image encoder, and three architectures were compared: DenseNet (densely connected convolutional network), ResNet (residual network), and SE-ResNet (squeeze-and-excitation residual network). The model comprised two output heads: a regression head for predicting two pulmonary function parameters—forced vital capacity (FVC) and forced expiratory volume in one second (FEV₁)—and a classification head for three-class differentiation of COPD, PRISm, and normal lung function. The results demonstrated that DenseNet achieved optimal performance within the multi-task learning framework, with an area under the receiver operating characteristic curve (AUC-ROC) of 0.85 and an overall accuracy of 0.79. For pulmonary function prediction, the highest concordance correlation coefficients for predicted values were 0.75 for FVC and 0.77 for FEV₁, respectively. In a subsequent study, Wu et al.[59] developed PulmoClass-3DAtt, a self-attention deep learning model for three-class classification of COPD, PRISm, and normal lung function. This study comprised 1,918 participants. Initially, a 3D fully convolutional network was employed as an encoder to extract lung parenchymal features from CT images, while concurrently predicting five pulmonary function parameters as an auxiliary task. The image features output by the encoder were subsequently fused with clinical variables and fed into a multi-head self-attention classifier based on the Transformer architecture for disease classification. In the three-class classification task, the model achieved an overall area under the receiver operating characteristic curve (AUC-ROC) of 0.86 and an accuracy of 0.87. Sensitivity for identifying COPD and normal lung function reached 0.90 and 0.88, respectively; however, sensitivity for PRISm was merely 0.42, whereas specificity was as high as 0.97, reflecting the substantial heterogeneity and atypical imaging manifestations of PRISm. This study represents the first application of self-attention mechanisms to the radiological classification of chronic respiratory diseases, enabling the capture of long-range spatial dependencies within lesions. Furthermore, through multi-task learning jointly optimizing pulmonary function prediction and disease classification, it establishes a novel technical paradigm for precise disease stratification and clinical decision support.

In summary, from 2024 to 2026, diverse AI methodologies—including exhaled metabolomics, blood-based environmental exposure assessment, dietary nutritional analysis, dual-phase and single-phase CT radiomics, multimodal deep learning, and self-attention Transformer architectures—have been systematically employed to identify PRISm-specific biomarkers and interpretable imaging features, collectively advancing research on early screening, precise subtyping, and risk prediction for PRISm. The development of distinct model categories may represent a promising direction for future research.

### 4.6. Data-driven intelligent analysis of pulmonary function

Traditional spirometry employs fixed diagnostic thresholds (e.g., FEV₁/FVC ≥ 0.7), whereas AI enhances diagnostic precision through dynamic threshold optimization and multidimensional parameter integration [60]. Operational errors during pulmonary function testing (PFT) may compromise data accuracy. Bonthada et al.[61] developed a deep learning system providing real-time feedback on flow-time/volume curve anomalies, achieving 93% accuracy in error detection and 94% precision in error subtype classification. Park et al.[62] established a CNN-based model predicting spirometric values from low-dose CT scans in 16,148 participants, demonstrating strong concordance (CCC = 0.94 for FVC, 0.91 for FEV₁) with mean absolute errors of 0.22L. This approach enables early identification of lung function decline.

Almeida et al. [63] quantified CT abnormalities to predict pulmonary function (AUC = 0.84) in the COPDGene cohort. A multicenter Japanese study [64] utilizing ConvNeXt CNN architecture on 130,000 chest X-rays achieved external validation correlation coefficients of 0.91–0.90 for FVC/FEV₁ estimation. This X-ray-based method proves particularly valuable for resource-limited settings given the modality's widespread availability. Complementary approaches combining radiomics with machine learning demonstrate comparable efficacy in automated PFT interpretation [65–67]. Collectively, these advancements underscore AI's transformative potential in PRISm-related lung function assessment. Lee et al.[68] employed a deep learning model to diagnose and stage COPD by predicting lung function indices, finding that a monophasic inspiratory CT model combined with clinical data (accuracy 65.2%–85.8%) performed comparably to a biphasic CT model (67.6%–88.0%) in COPD staging (GOLD staging), with the added benefit of significantly reducing patient radiation exposure. However, the study also revealed that PRISm patients (12% of the study population) were severely misclassified by all models (sensitivity only 19.3%–37.1%), with most being incorrectly assigned to the healthy group (GOLD 0). This low recognition rate underscores AI’s limitations in capturing PRISm’s unique pathological features, suggesting the need for complementary methods to prevent missed diagnoses. Future work should focus on elucidating imaging morphological differences between PRISm and GOLD 0 and optimizing the model’s discriminatory power to enable early intervention.

AI enhances the simplicity and accuracy of traditional PFT. AI-based pulmonary imaging demonstrates particular value as a spirometry alternative in clinical scenarios where conventional respiratory assessments are unavailable. This technological integration enables earlier diagnostic identification and therapeutic intervention in PRISm populations while reducing healthcare expenditures and mitigating regional disparities in respiratory care access.

### 4.7. Disease progression prediction

PRISm patients represent a clinically heterogeneous cohort exhibiting dynamic disease evolution trajectories, with three potential clinical endpoints: spirometric normalization, functional stabilization, or progression to airflow-obstructive COPD. This prognostic variability mandates systematic longitudinal surveillance and risk stratification, as timely therapeutic interventions may decelerate COPD pathogenesis in predisposed individuals. While extant predictive models predominantly focus on COPD progression, Wu et al.[69] developed the inaugural PRISm-specific prognostic framework utilizing electronic medical records from 283 patients (149 non-progressors vs 134 COPD converters over 24-month follow-up). LASSO-optimized multivariate logistic modeling identified six progression determinants: advanced age, dyspnea severity (mMRC ≥ 2), reduced FEV₁% predicted, diminished FEV₁/FVC ratio, concomitant respiratory comorbidities, and elevated erythrocyte counts. The model demonstrated robust discriminative capacity with AUCs of 0.87 (training cohort) and 0.79 (validation cohort), establishing China's first real-world clinical prediction tool for PRISm-to-COPD transition. This paradigm enables preemptive identification of high-risk subgroups for targeted intervention, with tobacco exposure burden, demographic factors, and ethnic predisposition identified as progression modifiers.

Although AI applications in PRISm progression research remain in the exploratory phase, contemporary methodological innovations lay essential groundwork for longitudinal mechanistic investigations and predictive model refinement.

### 4.8. Genomic research

Prior genomic investigations into PRISm have delineated potential pathophysiological pathways. A UK Biobank genome-wide association study (GWAS) [70] identified 22 single nucleotide polymorphism (SNP) loci attaining genome-wide significance (p < 5 × 10 ⁻ ⁸), predominantly colocalizing with diabetes-related genomic regions. Notably, four novel loci—rs7652391 (MECOM), rs9431040 (HLX), rs62018863 (TMEM114), and rs185937162 (HLA-B)—exhibited unprecedented associations with pulmonary functional decline, representing the inaugural GWAS establishing PRISm-specific genetic signatures. These findings emphasize the critical need for expanded spirometric phenotyping in respiratory genomic research.

AI integration in PRISm genomics remains underdeveloped, yet demonstrates transformative potential through accelerated GWAS pipelines via automated functional variant prioritization, individualized risk stratification through polygenic risk score optimization, and multi-omics network analysis elucidating disease endotypes. AI-enhanced genomic frameworks may transition conventional data mining paradigms toward clinically actionable insights, potentially enabling precision therapeutic closed-loop systems. Persistent challenges encompass heterogeneous data harmonization (κ < 0.6 across cohorts), limited model biological interpretability, and translational implementation barriers in clinical workflows.

## 5. Discussion

### 5.1. Strengths and limitations

The strength of this review lies in its unique focus on comprehensively evaluating AI technologies applied to PRISm, examining current advancements, limitations, and future directions to guide subsequent research developments. These studies suggest that future AI research on PRISm should prioritize three key domains: (i) multimodal data fusion to capture heterogeneous features; (ii) hybrid exposure modeling and multi-task learning architectures to enhance statistical robustness and learning efficiency; and (iii) interpretability methods and class imbalance correction to ensure clinical applicability and model reliability.

The primary limitation of this review is the small number of included studies, which permitted only a narrative synthesis of the findings. This paper endeavours to utilise relevant research to the fullest extent possible to compensate for this deficiency. Furthermore, the included studies were exclusively diagnostic in nature, lacking investigations into additional domains such as progression prediction and phenotyping. Future research should aim to provide robust evidence regarding the advantages and challenges of integrating AI into PRISm management. Finally, this review did not explore the rationale underlying model selection or the determinants of performance variations, as the optimal model may differ across distinct data types. Future investigations are anticipated to address this limitation and provide actionable guidance for practitioners seeking to implement AI in real-world clinical settings.

### 5.2. Challenges

#### 5.2.1. Data quality and standardization.

Currently, there remains a paucity of large-scale public datasets exclusively targeting PRISm populations. This scarcity potentially stems from inadequate recognition of the condition and persistent concerns regarding data security/privacy preservation. The majority of AI-driven PRISm investigations currently depend on institution-specific datasets or derivative subsets from COPD cohorts. Since the formal nosological definition of PRISm was only established in the 2023 Global Initiative for GOLD guidelines, extant datasets exhibit dual limitations: insufficient sample sizes and heterogeneous diagnostic criteria. The translational validity of AI-derived models necessitates rigorous methodological validation. Existing scholarship has predominantly concentrated on diagnostic differentiation challenges. However, growing clinical recognition and the establishment of standardized multicenter repositories are anticipated to catalyze systematic investigations, with predictive models achieving substantial performance optimization.

#### 5.2.2. Model explainability and clinical trust.

The “black-box” nature of AI models, characterized by opaque decision-making processes, impedes clinical translation and undermines clinical trust [71,72]. The emerging field of explainable artificial intelligence (XAI) addresses these limitations by prioritizing algorithmic transparency and interpretability alongside predictive accuracy [73–75]. A Belgian multicenter study [76] demonstrated that XAI-assisted pulmonary function test interpretation enhanced pulmonologists’ diagnostic accuracy by 10.4% (primary diagnosis) and 9.4% (differential diagnosis), while improving diagnostic confidence and inter-rater agreement. XAI methodologies encompass four principal approaches: Local explanations (e.g., Local Interpretable Model-agnostic Explanations) interpreting individual predictions; Global explanations (e.g., decision tree visualization) elucidating overall model behavior; Intrinsically interpretable architectures (e.g., linear regression) with inherent transparency; and Post-hoc explanations (e.g., Shapley Additive Explanations) providing retrospective model interpretation. SHAP values, rooted in cooperative game theory, quantify feature contributions through axiomatic value allocation [77–79]. Though conceptualized in 1953, their machine learning implementation emerged in 2010 [80], with Lundberg et al.[81] subsequently unifying SHAP with complementary techniques (LIME, DeepLIFT) within an integrated framework. Visualization techniques including heatmap analysis and decision-boundary mapping further enhance model interpretability [82–84]. Grad-CAM applications demonstrate particular utility in highlighting critical pulmonary features during convolutional neural network training [85]. However, methodological constraints persist: Grad-CAM may compromise model stability during backpropagation, while SHAP occasionally exhibits feature misprioritization [86]. XAI proves indispensable for error correction, trust establishment, and interdisciplinary innovation. Future investigations should refine temporal interpretability in longitudinal predictions while addressing current technical limitations. For PRISm research, both AI implementation and XAI integration remain nascent, warranting systematic exploration.

#### 5.2.3. Ethical and privacy considerations.

Patient health data persists in fragmented data silos across disparate healthcare institutions and research facilities, creating substantial barriers to centralized governance. Medical data sharing entails inherent privacy breach vulnerabilities, whereas excessive regulatory constraints may inadvertently compromise algorithmic efficacy. Tiered de-identification protocols implement context-sensitive masking strategies through dynamic data anonymization. During model optimization, differential privacy frameworks employ stochastic noise injection to ensure individual non-identifiability, though such approaches incur significant computational overhead and may induce algorithmic instability risks.

Federated learning (FL) architectures establish cryptographic model interactions, enabling multi-institutional knowledge transfer via encrypted parameter exchanges without raw data disclosure [87,88]. Originally conceptualized by Google in 2016 for edge device intelligence, FL has evolved into three principal paradigms: horizontal, vertical, and transfer FL. Despite facilitating cross-center collaborative modeling, FL implementations confront persistent challenges including computational overhead constraints, cross-site data heterogeneity (Cohen's d > 0.8), and adversarial attack vulnerabilities [89–91]. Current research prioritizes computational acceleration through homomorphic encryption optimization and federated robustness enhancement. Progressive resolution of ethical data stewardship dilemmas will likely catalyze establishment of large-scale PRISm-specific repositories. This evolution promises to ameliorate multi-modal healthcare data interoperability while advancing AI model performance through enhanced federated knowledge distillation.

### 5.3. Future perspectives

Although previous AI research on PRISm has spanned multiple domains—including respiratory metabolomics, environmental exposure assessment, radiomics, and deep learning—it has converged toward a unified future direction: the development of a precision prediction system integrating multi-source heterogeneous data, accounting for longitudinal dynamic changes, and providing clinical interpretability. Current studies have validated the significant advantages of multimodal fusion at the cross-sectional level; however, given that PRISm represents a highly heterogeneous transitional state, future AI models must evolve from static identification toward dynamic prediction. This necessitates prospective integration of imaging and pulmonary function data across multiple follow-up visits, utilizing time-series models or survival analysis frameworks to capture critical transition points and rates at which individuals progress from normal lung function through PRISm to COPD or undergo reverse transitions, thereby achieving genuine early warning of disease trajectories. Regarding data dimensions, molecular-level information—including the gut microbiome, blood transcriptome, and proteome—has yet to be fully integrated into existing models, beyond CT images and conventional clinical variables. Multi-level joint modeling of these omics features with imaging phenotypes is anticipated to elucidate mechanistic differences underlying distinct PRISm subtypes, thereby providing a biological foundation for personalized interventions. Methodologically, although radiomics and convolutional neural networks have demonstrated favorable performance, the potential of Transformer self-attention architectures in capturing long-range spatial dependencies across the entire lung has only begun to emerge. In the future, integrating large-scale pre-trained models with few-shot learning techniques is expected to overcome bottlenecks associated with limited PRISm samples and annotation challenges. Ultimately, the prerequisite for any advanced algorithm to achieve clinical adoption is that its decision-making process must be comprehensible and trustworthy to pulmonologists and radiologists. Consequently, developing more transparent visualization and interpretability tools, establishing user-friendly online interactive platforms, and proactively deploying models in clinical settings for real-world validation will constitute indispensable steps for this field to transition from technological exploration to clinical implementation.

## 6. Conclusion

This review systematically synthesizes recent investigations. Despite variations in data modalities and algorithmic strategies, these studies converge on a central premise: unidimensional assessment is insufficient for the early detection of PRISm in clinical practice. Effective characterization of this transitional phenotype necessitates the integration of multi-source heterogeneous data and the leveraging of deep learning for advanced feature extraction. The multidimensional AI research on PRISm encompasses diverse domains, including exhaled metabolic profiling, internal exposure to environmental pollutants, dietary and nutritional factors, and chest CT imaging analysis. Existing studies have established the feasibility of AI applications in cross-sectional contexts; nevertheless, constrained sample sizes, inadequate external validation, and limited model interpretability constitute significant barriers to clinical translation. Future investigations should prioritize the establishment of longitudinal cohorts to characterize the dynamic progression of PRISm, the deep integration of multi-scale imaging, molecular, and clinical data, and the adoption of transparent learning architectures to facilitate the development of precise risk stratification systems. Collectively, artificial intelligence is fundamentally transforming our understanding of PRISm, heralding unprecedented opportunities for early detection and personalized therapeutic interventions.

## Supporting information

S1 File Prisma 2020 checklist. (DOCX)

S2 File Search. (DOCX)
