# Supplementary material for: Comprehensive application of artificial intelligence in preserved ratio impaired spirometry: A systematic literature review
Source: PLoS One. 2026 Sep 16;21(9):e0353021. doi: 10.1371/journal.pone.0353021 (PMC13581010; doi:10.1371/journal.pone.0353021)
Supplement: Supplementary file 2 [file pone.0353021.s002.docx]

-2026-03-15-

PRISm

Preserved ratio impaired spirometry

Mesh: Deep Learning

Deep Learning

Learning, Deep

Hierarchical Learning

Learning, Hierarchical

Mesh: Machine Learning

Machine Learning

Learning, Machine

Transfer Learning

Learning, Transfer

Mesh: Radiomics

Radiomics

Radiomic

**PUBMED:**

| ID | Search | Results |
| --- | --- | --- |
| 1 | ((PRISm[Title/Abstract]) OR (Preserved ratio impaired spirometry[Title/Abstract])) AND ((((((("Deep Learning"[Mesh]) OR (Learning, Deep[Title/Abstract])) OR (Hierarchical Learning[Title/Abstract])) OR (Learning, Hierarchical[Title/Abstract])) OR (Deep Learning[Title/Abstract])) OR ((((("Machine Learning"[Mesh]) OR (Machine Learning[Title/Abstract])) OR (Learning, Machine[Title/Abstract])) OR (Transfer Learning[Title/Abstract])) OR (Learning, Transfer[Title/Abstract]))) OR ((("Radiomics"[Mesh]) OR (Radiomics[Title/Abstract])) OR (Radiomic[Title/Abstract]))) | **139** |

**WOS:**

| ID | Search | Results |
| --- | --- | --- |
| 1 | AB=(PRISm OR Preserved ratio impaired spirometry) AND ((TS=(Deep Learning) OR AB=(Learning, Deep OR Hierarchical Learning OR Learning, Hierarchical OR Deep Learning)) OR (TS=(Machine Learning) OR AB=(Machine Learning OR Learning, Machine OR Transfer Learning OR Learning, Transfer)) OR (TS=(Radiomics) OR AB=(Radiomics OR Radiomic))) | **230** |

**Cochrane library:**

| ID | Search | Results |
| --- | --- | --- |
| #1 | MeSH descriptor: [Deep Learning] explode all trees | 409 |
| #2 | (Deep Learning or Learning, Deep or Hierarchical Learning or Learning, Hierarchical):ti,ab,kw (Word variations have been searched) | 2414 |
| #3 | #1 or #2 | 2430 |
| #4 | MeSH descriptor: [Machine Learning] explode all trees | 1266 |
| #5 | (Machine Learning or Learning, Machine or Transfer Learning or Learning, Transfer):ti,ab,kw (Word variations have been searched) | 6265 |
| #6 | #4 or #5 | 6648 |
| #7 | MeSH descriptor: [Radiomics] explode all trees | 89 |
| #8 | (Radiomics or Radiomic):ti,ab,kw (Word variations have been searched) | 856 |
| #9 | #7 or #8 | 856 |
| #10 | (PRISm or Preserved ratio impaired spirometry):ti,ab,kw (Word variations have been searched) | 1644 |
| #11 | #3 or #6 or #9 | 8861 |
| #12 | #10 and #11 | **6** |

**Embase:**

| ID | Search | Results |
| --- | --- | --- |
| #1 | 'learning, deep':ab,ti OR 'hierarchical learning':ab,ti OR 'learning, hierarchical':ab,ti OR 'deep learning':ab,ti | 109,901 |
| #2 | 'machine learning':ab,ti OR 'learning, machine':ab,ti OR 'transfer learning':ab,ti OR 'learning, transfer':ab,ti | 203,690 |
| #3 | radiomics:ab,ti OR radiomic:ab,ti | 23,873 |
| #4 | #1 OR #2 OR #3 | 305,267 |
| #5 | prism:ab,ti OR 'preserved ratio impaired spirometry':ab,ti | 26,904 |
| #6 | #4 AND #5 | **195** |

**Scopus:**

| ID | Search | Results |
| --- | --- | --- |
| 1 | ( ( TITLE-ABS-KEY ( "PRISm" ) OR TITLE-ABS-KEY ( "Preserved ratio impaired spirometry" ) ) ) AND ( ( ( TITLE-ABS-KEY ( "Radiomics" ) OR TITLE-ABS-KEY ( "Radiomic" ) ) ) OR ( ( TITLE-ABS-KEY ( "Machine Learning" ) OR TITLE-ABS-KEY ( "Learning, Machine" ) OR TITLE-ABS-KEY ( "Transfer Learning" ) OR TITLE-ABS-KEY ( "Learning, Transfer" ) ) ) OR ( ( TITLE-ABS-KEY ( "Deep Learning" ) OR TITLE-ABS-KEY ( "Learning, Deep" ) OR TITLE-ABS-KEY ( "Hierarchical Learning" ) OR TITLE-ABS-KEY ( "Learning, Hierarchical" ) ) ) ) | **2007** |

**Ovid medline:**

| ID | Search | Results |
| --- | --- | --- |
| 1 | Deep Learning/ | 38030 |
| 2 | (Learning, Deep or Hierarchical Learning or Learning, Hierarchical or Deep Learning).ab,ti,kw. | 106833 |
| 3 | Machine Learning/ | 67560 |
| 4 | (Machine Learning or Learning, Machine or Transfer Learning or Learning, Transfer).ab,ti,kw. | 193509 |
| 5 | Radiomics/ | 3454 |
| 6 | (Radiomics or Radiomic).ab,ti,kw. | 18009 |
| 7 | (PRISm or Preserved ratio impaired spirometry).ab,ti,kw. | 15874 |
| 8 | 1 or 2 | 109502 |
| 9 | 3 or 4 | 200063 |
| 10 | 5 or 6 | 18011 |
| 11 | 8 or 9 or 10 | 289988 |
| 12 | 7 and 11 | **126** |
